# Supplementary material for: Change Point Detection in Correlation Networks
Source: Sci Rep. 2016 Jan 7;6:18893. doi: 10.1038/srep18893 (PMC4703970; doi:10.1038/srep18893)
Supplement: Supplementary Information [file srep18893-s1.pdf]

Change Point Detection in Correlation Networks: Supplementary Information

Ian Barnett
Department of Biostatistics, Harvard University
ibarnett@hsph.harvard.edu

Jukka-Pekka Onnela*
Department of Biostatistics, Harvard University
onnela@hsph.harvard.edu

1. APPENDIX: PROOF OF THEOREM 1

1.1 Proof of Theorem 1

The proof starts by writing the Frobenius norm as a sum over all pairs of observations, and then takes expectation, making use of what we know of the first two moments of a quadratic form of normally distributed variables. The following is the derivation of $E[d(k)]$ under H_0 for normally distributed observations:

$$
\begin{aligned}
d(k) = \;& \operatorname{tr}\{[S(1,k) - S(k+1,T)]^T [S(1,k) - S(k+1,T)]\} \\
= \;& \operatorname{tr}\{[S(1,k) - S(k+1,T)]^2\} \\
= \;& \operatorname{tr}\{(Y^T Y \underbrace{(D(1,k) - D(k+1,T))}_{C})^2\} \\
= \;& \textstyle\sum_{i=1}^T \sum_{j=1}^T (Y_i^T Y_j)^2 C_{jj} C_{ii} \\
= \;& \textstyle\sum_{i=1}^T \left\{ (Y_i^T Y_i)^2 C_{ii}^2 + \sum_{j \in \{1,\ldots,i-1,i+1,\ldots,T\}} (Y_i^T Y_j)^2 C_{ii} C_{jj} \right\}
\end{aligned}
$$

Taking expectation gives

$$
\begin{aligned}
E[d(k)] = \;& \textstyle\sum_{i=1}^T \left\{ E[(Y_i^T Y_i)^2] C_{ii}^2 + \sum_{j \in \{1,\ldots,i-1,i+1,\ldots,T\}} E[(Y_i^T Y_j)^2] C_{ii} C_{jj} \right\} \\
= \;& \left(\tfrac{1}{k} + \tfrac{1}{T-k}\right) E[(Y_i^T Y_i)^2] + \textstyle\sum_{i=1}^k \left(\tfrac{k-1}{k^2} - \tfrac{1}{k}\right) E[(Y_i^T Y_j)^2] \\
& + \textstyle\sum_{i=k+1}^T \left(\tfrac{T-k-1}{(T-k)^2} - \tfrac{1}{T-k}\right) E[(Y_i^T Y_j)^2] \\
= \;& \left(\tfrac{1}{k} + \tfrac{1}{T-k}\right) E[(Y_i^T Y_i)^2] + k \left(\tfrac{k-1}{k^2} - \tfrac{1}{k}\right) E[(Y_i^T Y_j)^2] \\
& + (T-k) \left(\tfrac{T-k-1}{(T-k)^2} - \tfrac{1}{T-k}\right) E[(Y_i^T Y_j)^2] \\
= \;& \left(\tfrac{1}{k} + \tfrac{1}{T-k}\right) E[(Y_i^T Y_i)^2] + \left(\tfrac{k-1}{k} - 1 + \tfrac{T-k-1}{T-k} - 1\right) E[(Y_i^T Y_j)^2] \\
= \;& \left(\tfrac{1}{k} + \tfrac{1}{T-k}\right) (2\operatorname{tr}(\Sigma^2) + \operatorname{tr}(\Sigma)^2) + \left(\tfrac{k-1}{k} + \tfrac{T-k-1}{T-k} - 2\right) \operatorname{tr}(\Sigma^2) \\
= \;& \left(\tfrac{1}{k} + \tfrac{1}{T-k}\right) (\operatorname{tr}(\Sigma^2) + \operatorname{tr}(\Sigma)^2)
\end{aligned}
$$

The last line is the result of Theorem 1.
